# CrossQuery: A Web Tool for Easy Associative Querying of Transcriptome Data

**DOI:** 10.1371/journal.pone.0028990

**Published:** 2011-12-12

**Authors:** Toni U. Wagner, Andreas Fischer, Eva C. Thoma, Manfred Schartl

**Affiliations:** 1 Physiological Chemistry I, Biocenter, University of Würzburg, Würzburg, Germany; 2 Vascular Biology and Tumor Angiogenesis, Medical Faculty Mannheim, Heidelberg University, Heidelberg, Germany; 3 Vascular Oncology and Metastasis, German Cancer Research Center Heidelberg (DKFZ-ZMBH Alliance), Heidelberg, Germany; University of Toronto, Canada

## Abstract

Enormous amounts of data are being generated by modern methods such as transcriptome or exome sequencing and microarray profiling. Primary analyses such as quality control, normalization, statistics and mapping are highly complex and need to be performed by specialists. Thereafter, results are handed back to biomedical researchers, who are then confronted with complicated data lists. For rather simple tasks like data filtering, sorting and cross-association there is a need for new tools which can be used by non-specialists. Here, we describe CrossQuery, a web tool that enables straight forward, simple syntax queries to be executed on transcriptome sequencing and microarray datasets. We provide deep-sequencing data sets of stem cell lines derived from the model fish Medaka and microarray data of human endothelial cells. In the example datasets provided, mRNA expression levels, gene, transcript and sample identification numbers, GO-terms and gene descriptions can be freely correlated, filtered and sorted. Queries can be saved for later reuse and results can be exported to standard formats that allow copy-and-paste to all widespread data visualization tools such as Microsoft Excel. CrossQuery enables researchers to quickly and freely work with transcriptome and microarray data sets requiring only minimal computer skills. Furthermore, CrossQuery allows growing association of multiple datasets as long as at least one common point of correlated information, such as transcript identification numbers or GO-terms, is shared between samples. For advanced users, the object-oriented plug-in and event-driven code design of both server-side and client-side scripts allow easy addition of new features, data sources and data types.

## Introduction

DNA sequencing techniques are rapidly evolving. It can be foreseen that in the next few years whole genome or exome sequencing will not only be frequently performed in research laboratories, but this technique will certainly be employed for diagnostic approaches in medicine. Next generation sequencing and microarray technologies produce enormous amounts of data for each single experiment. After raw analysis, normalization and bioinformatic filtering, researchers are provided with large data sets, which allow complex analyses [1], [2]. However, many experiments do not require such complicated analyses. Instead, researchers often want to answer rather simple questions such as comparing absolute or relative expression between or within data sets or the association with GO-terms [3]. To facilitate such operations we have developed a software tool that can be employed by non-specialists. CrossQuery enables easy filtering, sorting and cross-association of data. There are already a number of prominent web-tools and commercial programs for expression and deep sequencing analyses available (e.g. ArrayVision (GE Healthcare), Base (Lund University) [4], Bioconductor (www.bioconductor.org), Chipster (http://chipster.csc.fi/) [5], CLC Genomics Workbench (CLC bio), Expression Profiler (European Bioinformatics Institute) [6], GeneSifter (PerkinElmer, geospiza) [7], GeneXplorer (Stanford University) [8], GeneSpring (Agilent Technologies), J-Express (http://jexpress.bioinfo.no/site/) [9], Mayday (www.zbit.uni-tuebingen.de/pas/mayday) [10], Mayday SeaSight [11], Partek® Genomics Suite™ (Partek Inc.), SAM (www-stat.stanford.edu/~tibs/SAM/) [12], TM4 (www.tm4.org/) [13], XCluster (http://fafner.stanford.edu/~sherlock/cluster.html). However, their focus is either on very simple, commonly used pre-set analysis (like fold-change) or they are rather complex to manipulate. Many cannot be installed locally or only with costly licenses.

Most available tools are focused on cancer studies, mammalian data (human and mouse especially) and microarray-type data. Automated algorithms for analyses will only work on equalized datasets, which in turn are hard to produce with different techniques or over longer time spans and independent experiments. Hence, our goal was to develop a tool to complement already available ones. Therefore, emphasis was placed on simplicity, compatibility with any kind of quantitative data, freedom of query manipulation and the possibility to locally install and even extend or change the source code.

## Results and Discussion

We developed CrossQuery as a user-friendly tool for the filtering and sorting of normalized deep-sequencing and microarray data. CrossQuery provides an AJAX-based web-interface that allows users to basically ask questions using intuitive (MySQL-based) query syntax. Thanks to the pre-joined database tables and the AJAX-based system-architecture queries are returned within a few seconds and no page-refreshing or reloads are necessary. The modular, plug-in and event-driven design of both server and client-side scripts allow advanced users to easily add functions.

**Figure 1:**
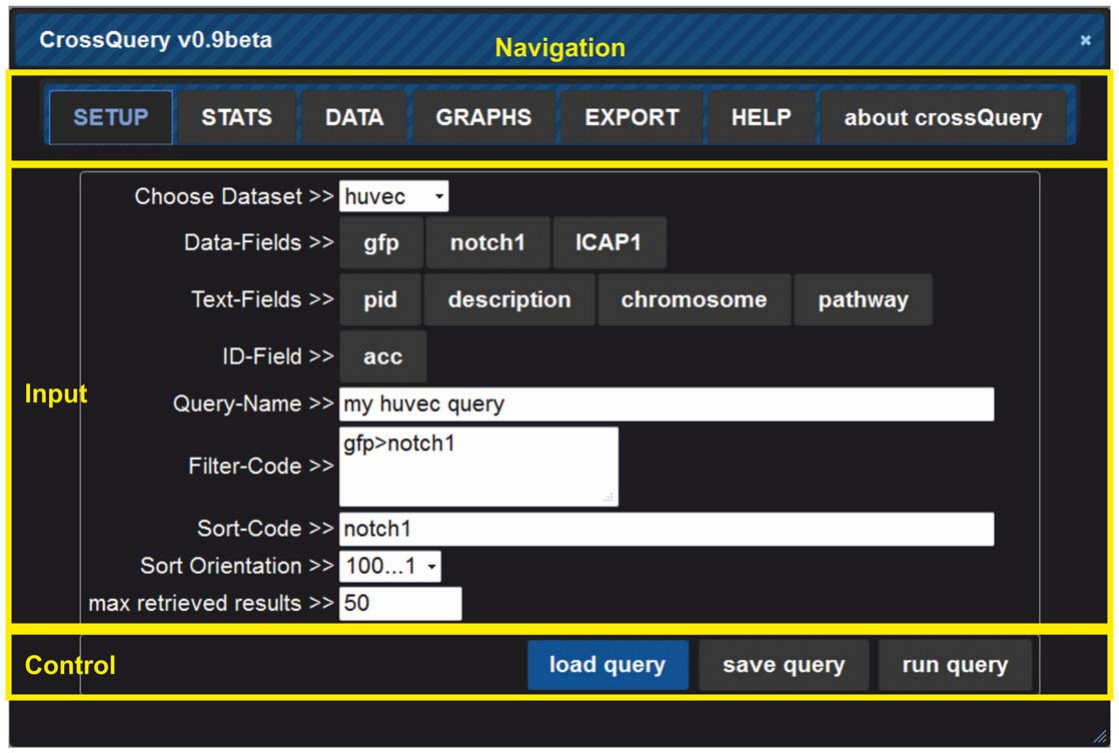
The general interface of CrossQuery employs a top-tab navigation to jump between the possible actions (yellow box labelled "Navigation"). The here shown "SETUP" tab allows to edit the query-settings ("Input" box). The box highlighted on the bottom ("Control") features three buttons that will run the given query on the server or allow loading and saving queries for later use.

Once logged in, CrossQuery is displayed as a tabbed window, which allows users to freely write and send queries to the server backend via the query interface (**Figure 1**). First, the user has to select the datasets to be worked with. When using the publicly available CrossQuery server, users are restricted to the provided datasets. These will be constantly extended and it will be possible to integrate datasets from other groups upon request. However, as CrossQuery can be installed platform-independently, researchers can easily set up their own server with any number of data tables. Therefore, users can upload their datasets, which had been normalized before.

The query syntax available to the users represents a variation of the MySQL select syntax. A short reference is available in the online help system. To open the system to inexperienced users, we chose to divide each query into three independent parameters: *FILTER*, *SORTER*, and *MAXROWS*. Several example queries are available via the help tab and listed in **Table 1**.

**Table 1:** CrossQuery syntax examples.

	task	formula	actual example
**Mathematical rules**	simple	SampleA > SampleB	sg3>mes1
	typical fold	SampleA/SampleB >10	sg3/mes1>10
**Coupling of subrules**	AND	SampleA > SampleB AND Sample B > SampleC Thresholding: SampleA/SampleB >10 AND SampleB >5	sg3>mes1 and mes1>dmrt1binding sg3/mes1>10 and mes1>5
	OR	SampleA > SampleB OR SampleB > SampleC	sg3>mes1 or mes1> dmrt1binding
	mixed	(SampleA>SampleB OR SampleA>200) AND (SampleC>20 AND SampleD<10)	(sg3>mes1 OR sg3>200) AND (dmrt1binding>20 AND eye<10)
**Logical NOT rules**	mathematical not equal	SampleA ! = SampleB	sg3! = mes1
**Text-based queries**	Searching in descriptions	find("a keyword you want to filter out",description)	find("hox",description)
	Searching in go-terms	find("go-term text", goterms)	find("dna binding",goterms)
	Searching for specific go-terms	find("go-term id", goterms)	find("3677",goterms)
	EXCLUDING keywords	NOT find("keyword",description)	NOT find("DNA",description)

The client-side script sends the user-provided data to the server (via an AJAX request) and waits for its answer. The server-side script then parses the query and executes it. On database level, all used datasets are pre-joined using their gene-ID and are then selected with the *FILTER* parameter extending the WHERE-clause of the MySQL select statement. The *SORTER* parameter is used to fill the ORDER BY clause of the MySQL select statement.

**Figure 2:**
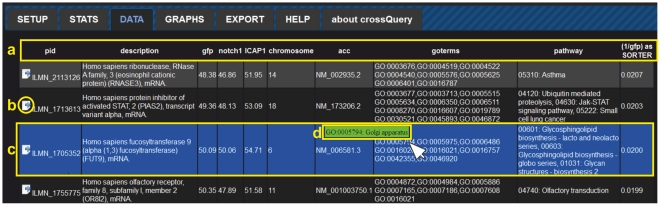
The Data tab of CrossQuery shows (**a**) **all columns of the dataset and adds an extra column (last one) to represent the value determined by the SORTER term defined during query setup.** (b) The export icon allows copying single data-rows to the export view. (c) The currently active row is always highlighted and clicking it will open the respective gene-sequence resource page (Ensembl or RefSeq). (d) The GO-term column supports displaying the text-representation of the GO-ID as a hover-effect.

**Figure 3:**
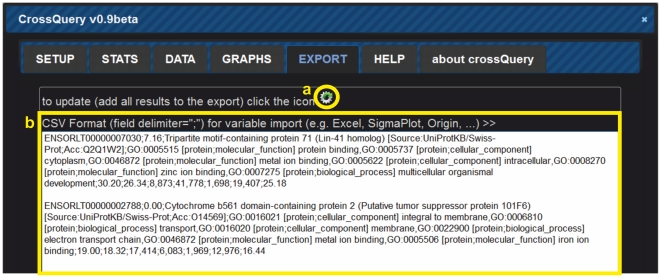
The Export tab of CrossQuery allows (**a**) **one-click export of all retrieved result-rows.** (b) The currently exported data-rows are shown in an editable text area as comma-separated-values.

**Figure 4:**
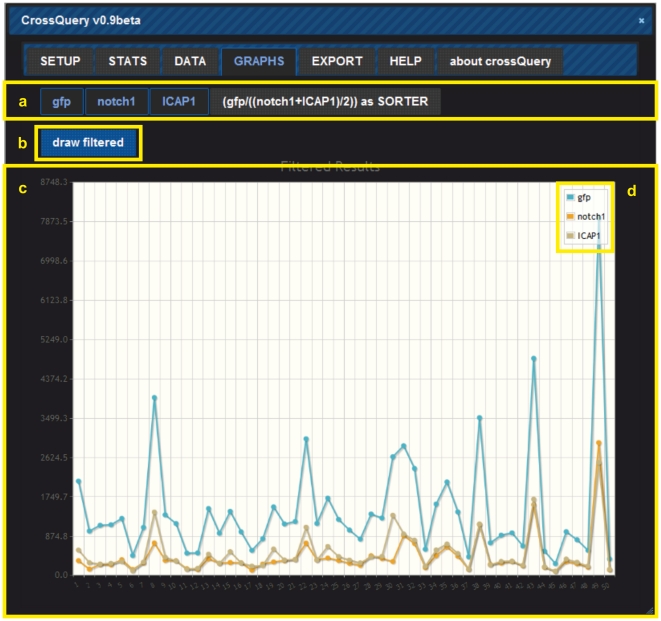
Using the Graphs tab of CrossQuery allows (**a**) **selection of data-columns to be rendered as lines in a plot.** (b) Clicking the "draw filtered" button will refresh the (c) line-graph view and the legend (d). Plotting is helpful when looking for general trends in different subsets of data, e.g. in the provided example all expression peaks are present in all lines ( = data-columns), but amplitudes and baseline expression differ strongly.

All the data retrieved from the database query are then returned to the client (in JSON format). The client-side script re-formats the received data into a table for display (**Figure 2**). Users can interact with their results, save the query, and export the results as comma-separated-values. This format is universally recognized by almost any data visualization, database and data-analyses programs. Interaction possibilities with each data-row include GO-term look-up, following links to corresponding Ensembl or RefSeq gene webpages, and (multi-)selection of individual rows for data export (**Figure 3**). Finally, we have added a plotting module (using the publicly available jQuery plugin jqPlot), which allows multi-line plotting of the filtered results (**Figure 4**).

CrossQuery enables scientists to quickly, easily and freely create any logical query. Thus, the wet-lab scientists who performed the original experiments are able to directly ask arrays of questions without the need for counseling bioinformaticians. The combinatory freedom of queries, especially the ability to define SORTERS and FILTERS independently, is unique amongst scientific query-tools and allows researchers to play in a trial and error manner with their data. This humanization of data-mining and analyses benefited two independent projects. We included the corresponding datasets, one derived from whole genome mRNA sequencing and the other from microarray analyses.

The first dataset is a typical example for one of the challenges connected to the unique possibilities that the new technologies of massive parallel sequencing bring along: the fact that such data will now be increasingly produced in non-mainstream model organisms, where bioinformatic support and genomic resources are less advanced than for instance for human, mice, zebrafish (Danio rerio) or the fruitfly Drosophila melanogaster.

One such emerging model is the small teleost fish Medaka (Oryzias latipes). As a model organism, Medaka is increasingly used to complement the zebrafish for analyses of early embryogenesis [14], but it has also been established as a toxicology model [15], cancer model [16] and - unique amongst fish models - it was possible to establish embryonic stem cell lines [17] and spermatogonial stem cell lines [18]. We included unpublished deep-sequencing data for both cells lines.

These deep-sequencing datasets were generated as a means to compare mRNA expression levels between two stem cell lines. The cell line *mes1* was chosen to represent Medaka embryonic stem cells [17], [19] and *sg3* to represent adult spermatogonial stem cells [18] with near-pluripotent differentiation potential [20]. We wanted to identify activating transcription factors that were co-regulated in the pluripotent cell lines mes1 and sg3. Most interesting were candidates with very low expression levels in both stem cell lines, as we were looking for genes that would be suitable for induction of differentiation. Using *"find('transcription factor',goterms) and find('activator',goterms)"* as the filter and inverted *"(sg3+mes1)"* as the sorter, the server returned the 213 out of 24662 matching data rows. Subsequently, the results were used in directed differentiation assays: factors were cloned into expression vectors, transfected into both cell lines and assayed for differentiation. For example, the second hit, MITF, led to successful targeted differentiation of both stem cell lines after ectopic expression of this transcription factor [20].

To demonstrate the ability of the system to incorporate various types of data into one query-system, we used a web-tool called RSAT [21] to find potential binding sites of the transcription factor DMRT1 in the Medaka genome. We then joined the new dataset to the transcriptome sequencing data-table using the transcript-id as common identifier. Consequently, it was possible to not only identify commonly regulated transcription factors, but to further filter them according to the likelihood of activation by a specific transcription factor, DMRT1, using the following filter term: *“find('transcription factor',goterms) AND find('activator',goterms) AND dmrt1binding>10”*.

Secondly, we provide microarray data of primary human endothelial cells from umbilical veins (HUVEC). HUVEC are the prototypic tool for endothelial cell research. Endothelial cells line all blood vessels and form a barrier between blood and the surrounding tissue. HUVEC can be genetically modified by RNAi or viral transduction and are thus ideally suited to determine the function of genes for the generation of blood vessels in vitro but also in vivo after transplantation into immunocompromized mice [22], [23], [24]. In the experiments performed to obtain the microarray results, HUVEC were adenovirally infected with GFP as control and integrin cytoplasmic domain associated protein-1 (ICAP1, also known as ITGB1BP1) or constitutive-active NOTCH1 (NOTCH1-icd) and processed for RNA extraction and microarray analysis 36 h post induction [23].

NOTCH1 and ICAP1-expressing HUVEC are impaired in forming blood vessels [23]. By filtering the data with CrossQuery using the term *"gfp>notch1 and gfp>ICAP1"* pro-angiogenic transcripts were found to be downregulated (17722/47767 of measured transcripts). Using the sort-term *"gfp/((notch1+ICAP1)/2)",* the top commonly regulated factors were listed first: ESM1 and MMP10. Both factors were consequently shown to be downstream of ICAP1 and NOCH1, which control endothelial proliferation and cell cycle [23].

## Methods

### Design and Implementation

CrossQuery (http://crossquery.labhive.com) is implemented on the server-side as a PHP script that reacts to POST queries by executing SQL-commands and responding in the JSON format. As database backend MySQL is employed, which is commonly used for biological databases, e.g. Ensembl. To optimize query performance, all datasets were pre-joined into one table. This allows very quick responses even on low-power CPU machines. The client-interface was written in JavaScript extended with help of the publicly available jQuery (http://www.jquery.com) framework and a series of plug-ins for that framework. By using the most common underlying systems (PHP, MySQL, JavaScript), the tool can be easily setup on any operating system. A README file explains setup, data import and adaption.

In principle, any server system can be used as long as a web-server with access to PHP and MySQL are installed. The database structure should be created by importing the provided sql-dump file. Most system parameters are set in the config.php file. The system imports data-sets from a dynamically extendable MySQL table. Other table-structures can be used, but need to be described in the config-file according to the provided example datasets.

The source-code of the complete project is publicly available at the OpenSource code-repository GitHub (git@github.com:itsatony/crossQuery.git).

User-management is controlled via the LabHive core system (also included in the GitHub source-code), which was also developed by our group. We opened registration on the CrossQuery site, but any group installing their own version can easily switch off registration in the PHP config file.

### Availability and Future Directions

Project name: CrossQuery

*Project home page:* http://crossquery.labhive.com

anonymous login is possible through guest:guest (login:password) or by registration

*Operation System:* Platform-independent

*Publicly available plugins/code used:* jQuery and its plugins jGritter, jQuery-UI, jQ-Plot, excanvas, jquery-md5

*Prerequisites:* Web Server (Apache recommended) with PHP support and MySQL support

MySQL Database server

*License:* Open Source (Gnu GPL)

*Source-code:* publicly available at https://github.com/varuul/labhive or using GIT-clients via git@github.com:itsatony/crossQuery.git

In future, we will first focus on building an import module for CSV-formatted expression level data. Then we would like to extend the underlying user-management system with an administration interface.

The core-system can be extended on both server- and client-sides by plugins. Currently, we advise external contributors to contact us, as there are no in-depth tutorials or automated generators. These tools are planned to be developed upon demand.
